# Overview of the National Institute on Aging (NIA)

**DOI:** 10.1093/geroni/igaf122.1554

**Published:** 2025-12-31

**Authors:** Richard Hodes

**Affiliations:** National Institute on Aging, Bethesda, Maryland, United States

## Abstract

Dr. Hodes will provide an overview of NIA, its research mission, and key NIA programs and initiatives.

